# A TLS-Motivated Non-Iterative Robust Square-Root Cubature Kalman Filter for Bearings-Only Tracking

**DOI:** 10.3390/s26113605

**Published:** 2026-06-05

**Authors:** Chaoqi Li, Hao Wu, Guoxu Zeng, Minbo Yang, Yijie Zhao, Ali Mehmood

**Affiliations:** Information and Navigation College, Air Force Engineering University, Xi’an 710077, China

**Keywords:** bearings-only tracking, square-root cubature Kalman filter, robust nonlinear filtering, sensor outliers, total least squares

## Abstract

**Highlights:**

**What are the main findings?**

- A TLS-motivated robust square-root cubature Kalman filter is developed for bearings-only tracking, integrating M-estimation into the SCKF through equivalent weighting while preserving the derivative-free, non-iterative square-root recursion.
- A closed-form three-piecewise weight function with statistically determined thresholds enables the principled down-weighting of moderate outliers and the complete rejection of extreme ones, with confidence-level-based thresholds that reduce scenario-dependent empirical tuning.

**What are the implications of the main findings?**

- The proposed RSCKF achieves the lowest position MSE and is the only tested filter whose ANEES remains within the customary Monte Carlo consistency band under severe measurement contamination.
- The non-iterative design maintains computational cost close to the standard SCKF, making the method suitable for real-time bearings-only tracking applications with intermittent sensor outliers.

**Abstract:**

Measurement outliers remain a major source of performance degradation in nonlinear bearings-only target tracking, where a few corrupted observations can produce large innovations and even trigger filter divergence. This paper proposes a non-iterative robust square-root cubature Kalman filter (RSCKF) for bearings-only tracking with measurement outliers. Motivated by a TLS-type errors-in-variables interpretation of the pseudo-linear bearings-only measurement model, robustness is introduced through a closed-form equivalent weighting and rejection mechanism within the square-root cubature Kalman filtering framework. The proposed method preserves the derivative-free square-root filtering structure in implementation and avoids inner fixed-point or variational iterations. For the scalar bearing update considered in this paper, the weighting thresholds are determined from a normalized innovation statistic using prescribed confidence levels, so that moderate outliers are down-weighted, and extreme ones are rejected. Simulations under nominal, moderately contaminated, and severely contaminated measurement conditions show that the proposed RSCKF achieves accuracy comparable to the standard square-root cubature Kalman filter (SCKF) in the Gaussian case, while providing improved robustness and only a small computational overhead under the measurement outliers. Under the most severe contamination setting, where 15% of the bearings are corrupted by outliers with a standard deviation 30 times the nominal noise level, RSCKF limits the position-MSE increase to 8.4% relative to the nominal case and achieves the lowest position MSE among the seven compared filters, whereas the standard SCKF deteriorates by more than two orders of magnitude. It is also the only filter whose time-averaged ANEES remains within the consistency band used in the Monte Carlo evaluation, with a computation time close to that of the baseline CKF.

## 1. Introduction

Nonlinear filtering has long been an important topic in sensor fusion, navigation, and target tracking [1,2]. Various approaches have been developed, including the extended Kalman filter (EKF) [3], deterministically sampled filters (DSFs), and particle filtering (PF) methods [4]. The EKF employs a Jacobian-based linearization of nonlinear system models, which can yield inaccurate predictions and may become numerically fragile in strongly nonlinear conditions. DSFs, such as the unscented Kalman filter (UKF) [5] and the cubature Kalman filter (CKF) [6], propagate a set of deterministically chosen sigma or cubature points to approximate the posterior density and are often more accurate and stable than the EKF. The square-root CKF (SCKF) further enhances numerical robustness by propagating the Cholesky factor of covariance, commonly via QR-based factor updates, which helps preserve positive definiteness throughout the filtering process [6]. However, this square-root implementation only improves numerical conditioning; it does not provide statistical robustness against outlying measurement innovations by itself.

Despite these advances, standard DSFs are typically derived under Gaussian noise assumptions. In practice, measurements are frequently contaminated by outliers arising from sensor faults, multipath effects, or intermittent interference. Such non-Gaussian contamination can generate unusually large innovations, leading to severe estimation degradation or even filter divergence. The PF can handle non-Gaussian noise through Monte Carlo integration, but it suffers from enormous computational cost and may break down when contamination is severe and sporadic [4].

Robust estimation provides an attractive alternative for mitigating outliers while retaining computational tractability. Huber’s M-estimation limits the influence of large residuals by exhibiting *l*_2_-norm properties for nominal measurements and transitioning toward *l*_1_-norm behavior for the contaminated ones [7]. However, the M-estimation was originally developed for linear systems [8], and its extension to nonlinear systems via EKF-type linearization may degrade accuracy in highly nonlinear conditions [9]. To address this issue, regression-based robust DSFs were developed by incorporating M-estimation into the UKF and the divided difference filter through linear or nonlinear regression formulations [10,11,12,13,14]. The nonlinear regression variants, such as the NRUKF [12], can improve robustness, but their algorithmic implementations require explicit Jacobian evaluations and iterative procedures, sacrificing the derivative-free computational advantage of DSFs, which increases computational burden. Sahl [15] proposed a Huber-based robust cubature Kalman filter for bearings-only tracking with missing measurements, but the algorithm framework was built on Gaussian noise assumptions and was difficult to apply in practice under non-Gaussian noise. Li [16] proposed a robust recursive sigma point Kalman filter for Huber-based generalized M-estimation, but the recursion depth must be preset and cannot be adapted online in practical engineering applications.

Recent studies have also emphasized adaptive and application-oriented nonlinear filtering. Hierarchical learning-based cascaded adaptive filtering has been developed for nonlinear system identification [17], and adaptive robust CKF variants have been proposed for maneuvering target tracking with model uncertainty and abnormal measurement noises [18].

Several alternative robust filtering paradigms have emerged in recent years. The maximum correntropy criterion (MCC) employs a Gaussian kernel similarity measure that naturally suppresses large residuals and can be combined with the CKF to yield a derivative-free robust filter [19]. However, its performance depends critically on a kernel bandwidth parameter that is difficult to tune across varying contamination levels [20,21]. Heavy-tailed probabilistic models offer another direction. Student’s *t*-based filters [22,23] and their Gaussian–Student’s *t* mixture extensions [24,25] adaptively inflate measurement uncertainty under large residuals, broadening noise-modeling flexibility at the cost of iterative variational Bayesian (VB) inference and sensitivity to degree-of-freedom hyperparameters. More generally, VB-based robust CKFs with outlier detection [26,27,28,29] can jointly estimate the state and unknown noise statistics, improving adaptivity under model mismatch, but the inner VB iterations introduce non-negligible computational overhead and additional tuning effort; when adaptive stopping is used, the average cost may further increase in heavily contaminated cases.

Bearings-only tracking (BOT) estimates the position and velocity of a target using bearing measurements, which is a representative nonlinear filtering problem [30]. Recent studies on angle-only filtering and tracking further show that bearings-only estimation remains important in passive sonar, passive radar, infrared surveillance, and other passive sensing applications [31]. BOT is particularly sensitive to measurement outliers because a single corrupted bearing can dominate the measurement update and trigger filter divergence. In this study, we propose a robust square-root cubature Kalman filter (RSCKF) for the BOT problem. Compared with the conventional robust filters, the main contributions are twofold.

1. M-estimation is integrated into the SCKF via a TLS- motivated cost function tailored to the BOT pseudo-linear EIV structure [32,33,34]. Robustness is achieved by replacing the prior measurement weight with an equivalent weight matrix, so that the standard SCKF recursion and square-root numerics are fully preserved without Jacobian evaluations, linearization, or iterative procedures.
2. A closed-form three-piecewise equivalent weight function is designed for the scalar bearing update. The standardized innovation serves as the outlier judgment variable, and its two thresholds are derived directly from the prescribed significance levels of the standard normal distribution, thereby reducing reliance on scenario-dependent empirical tuning. Normal measurements pass through unmodified, moderate outliers are down-weighted according to the minimum-intervention principle, and extreme outliers are rejected entirely. This complete rejection capability is absent from Huber’s method, where the weight remains non-zero regardless of the outlier magnitude.

The remainder of this paper is organized as follows. Section 2 reviews the system model and the standard SCKF. Section 3 derives the proposed RSCKF based on the TLS criterion and M-estimation. Section 4 presents the equivalent weight function design and implementation details. Section 5 provides numerical simulations and comparisons. Section 6 concludes the paper.

## 2. System Model and Square-Root Cubature Kalman Filter

We first present the nonlinear filtering model in a general form and then specialize it to the bearings-only tracking (BOT) problem considered in this paper. In a general nonlinear system, a known input or control signal may be present in the state transition. Thus, the model can be written as:

(1) $$x_{k}=f\left(x_{k-1},u_{k-1}\right)+v_{k-1}$$

(2) $$z_{k}=h\left(x_{k}\right)+e_{k}$$

where the state vector is $x_{k}\in {\mathbb{R}}^{n_{x}}$, and the measurement vector is $z_{k}\in {\mathbb{R}}^{n_{z}}$. The functions $f\left(\cdot \right)$ and $h\left(\cdot \right)$ denote the state transition function and the measurement function, respectively. Here $u_{k-1}$ denotes a known input when available. The process noise is denoted by $v_{k-1}$, and the measurement noise vector is $e_{k}={\left[e_{k,1},\cdots ,e_{k,n_{z}}\right]}^{T}$.

In the BOT problem, the state of the target is $x_{k}={\left[x_{k}^{t},y_{k}^{t},{\dot{x}}_{k}^{t},{\dot{y}}_{k}^{t}\right]}^{T}$, where $x_{k}^{t}={\left[x_{k}^{t},y_{k}^{t}\right]}^{T}$ is the position and ${\;\dot{x}}_{k}^{t}={\left[{\dot{x}}_{k}^{t},{\dot{y}}_{k}^{t}\right]}^{T}$ is the velocity at time index k (k=1,2,⋯,n). The state of the observer is $x_{k,i}^{o}={\left[x_{k,i}^{o},y_{k,i}^{o},{\dot{x}}_{k,i}^{o},{\dot{y}}_{k,i}^{o}\right]}^{T}$, where $i=1,2,\cdots ,n_{z}$. Specializing the above general nonlinear model to the BOT problem considered in this paper, the target dynamics are written in the unforced form because no known control input is applied to the target. The specific target-motion model used in the simulations is given later in Section 5. The BOT-specific measurement equation takes the bearing-angle form, and the system model can be written as:

(3) $$x_{k}=f\left(x_{k-1}\right)+v_{k-1}$$

(4) $$z_{k,i}=\arctan\left(\frac{x_{k}^{t}-x_{k,i}^{o}}{y_{k}^{t}-y_{k,i}^{o}}\right)+e_{k,i}$$

where $z_{k,i}$ is the *i*-th component of $z_{k}={\left[z_{k,1},z_{k,2},\cdots ,z_{k,n_{z}}\right]}^{T}$ at time index k, the noises $v_{k-1}$ and $e_{k}$ are typically assumed to be Gaussian, i.e., $v_{k}∼N\left(0,Q\right)$ and $e_{k}∼N\left(0,R\right)$. Equation (4) is the standard line-of-sight bearing-angle measurement model used in BOT, expressing the angle subtended by the target relative to the *i*-th observer. The known observer position $\left(x_{k,i}^{o},y_{k,i}^{o}\right)$ is explicitly included in this measurement equation. In the simulations, the bearing angle is evaluated with a quadrant correction to avoid ambiguity caused by the standard arctangent function.

The CKF employs spherical–radial cubature points to solve the nonlinear filtering problem. Its square-root form, namely the square-root CKF (SCKF), propagates square-root factors instead of covariance matrices, thereby improving numerical stability and accuracy [6].

In the SCKF framework, the cubature points are $\xi_{j}=\sqrt{n_{x}}{\left[\begin{array}{cc} I_{n_{x}} & -I_{n_{x}} \end{array}\right]}_{j}$ with the weights $1/2n_{x}$, where $I_{n_{x}}$ is the $n_{x}$-dimensional identity matrix, and ${\left[\cdot \right]}_{j}$ represents the $j^{th}$ column of $\left[\cdot \right]$.

### 2.1. Time Update

In the time update step, the cubature points are generated as:

(5) $$X_{j,k-1|k-1}=S_{k-1|k-1}\xi_{j}+{\hat{x}}_{k-1|k-1},j=1,2,\cdots ,2n_{x}$$

where ${\hat{x}}_{k-1|k-1}$ is the estimated state at time index k−1, and $S_{k-1|k-1}$ is the square-root factor of the estimated covariance $P_{k-1|k-1}$ such that $P_{k-1|k-1}=S_{k-1|k-1}S_{k-1|k-1}^{T}$.

Then, the cubature points are propagated through the state equation:

(6) $$X_{j,k|k-1}^{*}=f\left(X_{j,k-1|k-1}\right)$$

Accordingly, the predicted state ${\hat{x}}_{k|k-1}$ and the square-root factor of the predicted covariance $S_{k|k-1}$ are computed as:

(7) $${\hat{x}}_{k|k-1}=\frac{1}{2n_{x}}\sum_{j=1}^{2n_{x}}X_{j,k|k-1}^{*}$$

(8) $$S_{k|k-1}=\mathrm{qr}\left(\left[\chi_{k|k-1}^{*},S_{Q}\right]\right)$$

where $\mathrm{qr}\left(\cdot \right)$ represents the $n_{x}\times n_{x}$ lower triangular matrix obtained by the QR decomposition, and $Q=S_{Q}S_{Q}^{T}$. In practice, $\chi_{k,k-1}^{*}$ can be written as:

(9) $$\chi_{k|k-1}^{*}=\frac{1}{\sqrt{2n_{x}}}\left[X_{1,k|k-1}^{*}-{\hat{x}}_{k|k-1}\;,X_{2,k|k-1}^{*}-{\hat{x}}_{k|k-1},\cdots ,X_{2n_{x},k|k-1}^{*}-{\hat{x}}_{k|k-1}\right]$$

### 2.2. Measurement Update

For the measurement update step, the predicted cubature points are regenerated as:

(10) $$X_{j,k|k-1}=S_{k|k-1}\xi_{j}+{\hat{x}}_{k|k-1},j=1,2,\cdots ,2n_{x}$$

and then propagated through the measurement function:

(11) $$Z_{j,k|k-1}=h(X_{j,k|k-1}),j=1,2,\cdots ,2n_{x}$$

Accordingly, the predicted measurement is:

(12) $${\hat{z}}_{k|k-1}=\frac{1}{2n_{x}}\sum_{j=1}^{2n_{x}}Z_{j,k|k-1}$$

The Kalman gain is given by:

(13) $$K_{k}=\left(P_{xz,k|k-1}/{\left(S_{zz,k|k-1}\right)}^{T}\right)/S_{zz,k|k-1}$$

where “/” denotes solving the corresponding triangular systems rather than explicit matrix inversion. $P_{xz,k|k-1}$ is the cross-covariance matrix, and $S_{zz,k|k-1}$ is the square-root factor of the innovation covariance matrix:

(14) $$P_{xz,k|k-1}=\sum_{j=1}^{2n_{x}}\frac{1}{2n_{x}}({\hat{x}}_{k|k-1}-X_{j,k|k-1}){\left({\hat{z}}_{k|k-1}-Z_{j,k|k-1}\right)}^{T}$$

(15) $$S_{zz,k|k-1}=\mathrm{qr}\left(\left[\zeta_{k|k-1},S_{R}\right]\right)$$

where $\zeta_{k|k-1}$ is:

(16) $$\zeta_{k|k-1}=\frac{1}{\sqrt{2n_{x}}}\left[Z_{1,k|k-1}-{\hat{z}}_{k|k-1}\;,Z_{2,k|k-1}-{\hat{z}}_{k|k-1},\cdots ,Z_{2n_{x},k|k-1}-{\hat{z}}_{k|k-1}\right]$$

$S_{R}$ denotes a square-root factor of R such that $R=S_{R}S_{R}^{T}$.

Therefore, the estimated state ${\hat{x}}_{k|k}$ and the square-root factor of the estimated covariance $S_{k|k}$ can be updated as:

(17) $${\hat{x}}_{k|k}={\hat{x}}_{k|k-1}+K_{k}(z_{k}-{\hat{z}}_{k|k-1})$$

(18) $$S_{k|k}=\mathrm{qr}\left(\left[\chi_{k|k-1}-K_{k}\zeta_{k|k-1},\;K_{k}S_{R}\right]\right)$$

where

(19) $$\chi_{k|k-1}=\frac{1}{\sqrt{2n_{x}}}\left[X_{1,k|k-1}-{\hat{x}}_{k|k-1}\;,X_{2,k|k-1}-{\hat{x}}_{k|k-1},\cdots ,X_{2n_{x},k|k-1}-{\hat{x}}_{k|k-1}\right]$$

## 3. The M-Estimation-Based SCKF According to the TLS Criterion

Standard Kalman filtering relies on the assumption of Gaussian measurement noise. However, in practical engineering scenarios, measurements are often contaminated by outliers, resulting in a heavy-tailed distribution. This can be modeled as a Gaussian mixture distribution G:

(20) G=(1−ε)D+εM

where $D∼N\left(0,R\right)$ represents the nominal Gaussian distribution, M denotes the contaminating distribution (typically heavy-tailed) caused by outliers, and $\varepsilon \in \left[0,1\right]$ is the contamination rate. When ε>0, the standard squared-error loss function becomes suboptimal. To address this, we introduce the M-estimation technique combined with the total least squares (TLS) criterion to ensure robustness against both measurement outliers and linearization errors in the BOT problem.

### 3.1. TLS-Based Extremal Function for Bearings-Only Tracking

For a linear system where both (1) and (2) are linear, the corresponding least-squares (LS) extremal function

(21) $$\Omega =\min\left(r_{x_{k|k-1}}^{T}P_{k|k-1}^{-1}r_{x_{k|k-1}}+r_{k}^{T}R^{-1}r_{k}\right)$$

where min(⋅) represents the minimization operator, $r_{x_{k|k-1}}={\hat{x}}_{k|k}-{\hat{x}}_{k|k-1}$ is the predicted error vector, $P_{k|k-1}$ represents the predicted covariance, and $r_{k}$ represents the measurement residual vector.

Both the linear dynamic model and the linear measurement model can be represented by the least squares (LS) extremal function, commonly referred to as the LS-LS extremal function. The solution is obtained by taking the derivative of the extremal function in Equation (21).

In the BOT nonlinear problem, by introducing the observation vector ${\tilde{b}}_{k}\in {\mathbb{R}}^{n_{z}}$ and the coefficient matrix ${\tilde{H}}_{k}\in {\mathbb{R}}^{n_{z}\times 2}$, the nonlinear bearing measurement can be transformed into a pseudo-linear form without approximation [32]. Let ${\tilde{b}}_{k}={\left[{\tilde{b}}_{k,1},\cdots ,{\tilde{b}}_{k,n_{z}}\right]}^{T}$ and ${\tilde{H}}_{k}=\left[\cos{\tilde{z}}_{k},\;-\sin{\tilde{z}}_{k}\right]$, where ${\tilde{z}}_{k}={\left[{\tilde{z}}_{k,1},\cdots ,{\tilde{z}}_{k,n_{z}}\right]}^{T}$ and ${\tilde{b}}_{k,i}=x_{k,i}^{o}\cos{\tilde{z}}_{k,i}$$-y_{k,i}^{o}\sin{\tilde{z}}_{k,i}$. The transformed measurement equation is:

(22) $${\tilde{H}}_{k}{\left[x_{k}^{t},y_{k}^{t}\right]}^{T}={\tilde{b}}_{k}$$

Considering the presence of noise in the measurements, errors exist in both the observation vector and the coefficient matrix. Let $e_{b_{k}}={\left[e_{b_{k},1},\cdots ,e_{b_{k},n_{z}}\right]}^{T}$ and $E_{H_{k}}=\left[e_{H_{k}^{1}},e_{H_{k}^{2}}\right]$ respectively, such that $b_{k}={\tilde{b}}_{k}+e_{b_{k}}$ and $H_{k}={\tilde{H}}_{k}+E_{H_{k}}$. Consequently, Equation (22) can be rewritten as:

(23) $$H_{k}{\left[x_{k}^{t},y_{k}^{t}\right]}^{T}=b_{k}$$

That is, the measurement model is equivalently transformed into Equation (23) without approximation. Equation (23) constitutes an errors-in-variables (EIV) model, and the standard LS solution is biased because errors exist in both the observation vector $b_{k}$ and the coefficient matrix $H_{k}$. Therefore, the total least squares (TLS) criterion is adopted. The TLS cost function minimizes the weighted errors in both $b_{k}$ and $H_{k}$ [33,34]:

(24) $$\Psi =\min\left(e_{b_{k}}^{T}P_{b_{k}}^{-1}e_{b_{k}}+e_{H_{k}}^{T}P_{H_{k}}^{-1}e_{H_{k}}\right)$$

where $P_{b_{k}}$ is the covariance matrix of $b_{k}$, and $P_{H_{k}}$ is the covariance matrix of $H_{k}$. $e_{H_{k}}=\mathrm{vec}(E_{H_{k}})$ and “vec” denotes the operator that converts a matrix underneath the previous one. Since $e_{b_{k,i}}$, $e_{H_{k,i}^{1}}$, and $e_{H_{k,i}^{2}}$ are all driven by the same bearing noise $e_{k.i}$, their exact joint covariance contains cross-correlation terms. The TLS cost function in Equation (24) adopts a decoupled (block-diagonal) approximation by treating $e_{b_{k}}$ and $e_{H_{k}}$ as separately weighted. This approximation is justified by two considerations: (*i*) the first-order expansion in Equation (26) introduces $O(e_{k,i})$ coupling only, and the cross terms are second-order; (*ii*) in the subsequent robust weighting step (Section 3.2), the same scalar weight ${\bar{w}}_{k,i}$ is assigned to all error components from the *i*-th bearing channel, which implicitly re-couples the error contributions at the weighting level.

Combining the TLS cost with the state prediction error, the extremal function for BOT can be obtained as:

(25) $$\Omega =\min\left(r_{x_{k|k-1}}^{T}P_{k|k-1}^{-1}r_{x_{k|k-1}}+\left(e_{b_{k}}^{T}P_{b_{k}}^{-1}e_{b_{k}}+e_{H_{k}}^{T}P_{H_{k}}^{-1}e_{H_{k}}\right)\right)$$

Considering the first-order Taylor expansion, the error terms can be approximated using the bearing noise $e_{k,i}=z_{k,i}-{\tilde{z}}_{k,i}$, such that:

(26) $$\left\{\begin{array}{l} \sin z_{k,i}=\sin({\tilde{z}}_{k,i}+e_{k,i})=\sin{\tilde{z}}_{k,i}+e_{k,i}\cos{\tilde{z}}_{k,i}\\ \cos z_{k,i}=\cos({\tilde{z}}_{k,i}+e_{k,i})=\cos{\tilde{z}}_{k,i}-e_{k,i}\sin{\tilde{z}}_{k,i} \end{array}\right.$$

The observation vector error can be rewritten as $e_{b_{k},i}=\left(-y_{k,i}^{o}\cos{\tilde{z}}_{k,i}-x_{k,i}^{o}\sin{\tilde{z}}_{k,i}\right)e_{k,i}$. Accordingly, $P_{b_{k}}$ can be expressed by:

(27) $$P_{b_{k}}={\tilde{P}}_{b_{k}}R=\mathrm{diag}\left({\left(y_{k,i}^{o}\cos{\tilde{z}}_{k,i}+x_{k,i}^{o}\sin{\tilde{z}}_{k,i}\right)}^{2}R_{i}\right)$$

where $\mathrm{diag}\left(\cdot \right)$ is the operator that puts a vector or matrix on the main diagonal, and $R_{i}$ is the *i*-th diagonal component of R, $i=1,2,\cdots ,n_{z}$.

Similarly, $P_{H_{k}}$ is obtained by:

(28) $$P_{H_{k}}={\tilde{P}}_{H_{k}}R=\mathrm{diag}\left(\left[\begin{array}{cc} \sin^{2}{\tilde{z}}_{k,i} & \sin{\tilde{z}}_{k,i}\cos{\tilde{z}}_{k,i}\\ \sin{\tilde{z}}_{k,i}\cos{\tilde{z}}_{k,i} & \cos^{2}{\tilde{z}}_{k,i} \end{array}\right]R_{i}\right)$$

From Equations (27) and (28), it can be observed that both $P_{b_{k}}$ and $P_{H_{k}}$ share the common factor R. Specifically, $P_{b_{k}}={\tilde{P}}_{b_{k}}R$ and $P_{H_{k}}={\tilde{P}}_{H_{k}}⊗R$, where ${\tilde{P}}_{b_{k}}$ and ${\tilde{P}}_{H_{k}}$ is defined analogously from Equation (28) with $R_{i}$ factored out. Substituting these into Equation (24) and combining with the state prediction term yields:

(29) $$\Omega =\min\left(r_{x_{k|k-1}}^{T}P_{k|k-1}^{-1}r_{x_{k|k-1}}+R^{-1}\left(e_{b_{k}}^{T}{\tilde{P}}_{b_{k}}^{-1}e_{b_{k}}+e_{H_{k}}^{T}{\tilde{P}}_{H_{k}}^{-1}e_{H_{k}}\right)\right)$$

Corresponding to the LS-LS extremal function, Equation (29) can be called the LS-TLS extremal function. In general, seeking an optimal or analytical solution involved in Equation (29) is typically intractable. In this work, the SCKF is used as an efficient numerical solution framework.

### 3.2. Generalized M-Estimation and Incorporation into the SCKF

Although the LS-TLS extremal function optimally handles the EIV model under purely Gaussian noise, its quadratic nature makes it vulnerable to the outlier component defined in Equation (20). To mitigate the impact of outliers, we replace the quadratic loss term with a robust score function $\rho \left(\cdot \right)$, transforming the problem into a generalized M-estimation framework.

The error equation associated with the estimated state ${\hat{x}}_{k|k}$ can be described as:

(30) $$v_{k}=H_{k}{\hat{x}}_{k|k}-b_{k}$$

If the coefficient matrix $H_{k}$ is exact, the error $v_{k}$ originates solely from $b_{k}$. The M-estimation criterion can be formulated as:

(31) $$\Omega =\min\left(\frac{1}{2}r_{x_{k|k-1}}^{T}P_{k|k-1}^{-1}r_{x_{k|k-1}}+\sum_{i=1}^{n_{z}}R_{i}^{-1}\rho (r_{k,i})\right)$$

Classical Huber-type M-estimation limits the influence of large residuals by using a bounded score function, but it does not reject them completely. In this paper, the M-estimation idea is used in an equivalent-weighting sense, and the robust measurement update is defined directly by a closed-form weight. This permits the three-region rule in Section 4 to down-weight moderate innovations and reject extreme ones, while preserving the same square-root cubature implementation.

In practice, $H_{k}$ is also contaminated by noise, hence the TLS-consistent robust extremal function is adopted:

(32) $$\Omega =\min\left(\frac{1}{2}r_{x_{k|k-1}}^{T}P_{k|k-1}^{-1}r_{x_{k|k-1}}+\sum_{i=1}^{n_{z}}R_{i}^{-1}\rho \left(e_{b_{k,i}},e_{H_{k,i}}\right)\right)$$

Minimizing Ω with respect to ${\hat{x}}_{k|k}$ leads to the introduction of weight factors. Taking the derivative of Equation (32) and setting it to zero yields:

(33) $$\begin{array}{l} \frac{\partial \Omega }{\partial {\hat{x}}_{k|k}}=P_{k|k-1}^{-1}r_{x_{k|k-1}}+\sum_{i=1}^{n_{z}}R_{i}^{-1}\left[\left(\frac{\partial e_{b_{k,i}}}{\partial {\hat{x}}_{k|k}}\right)\varphi_{1}\left(e_{b_{k,i}}\right)+\right.\\ \left.\left(\frac{\partial e_{H_{k,i}^{1}}}{\partial {\hat{x}}_{k|k}}\right)\varphi_{2}\left(e_{H_{k,i}^{1}}\right)+\left(\frac{\partial e_{H_{k,i}^{2}}}{\partial {\hat{x}}_{k|k}}\right)\varphi_{3}\left(e_{H_{k,i}^{2}}\right)\right]=0 \end{array}$$

where $\varphi_{i}\left(\cdot \right)$ denotes the derivative of $\rho \left(\cdot \right)$.

To handle outliers, weight factors are introduced for the contaminated measurements. By substituting $\varphi_{1}\left(w_{bi}e_{b_{k,i}}\right)$, $\varphi_{2}\left(w_{bi}e_{H_{k,i}^{1}}\right)$, and $\varphi_{3}\left(w_{bi}e_{H_{k,i}^{2}}\right)$ for $\varphi_{1}\left(e_{b_{k,i}}\right)$, $\varphi_{2}\left(e_{H_{k,i}^{1}}\right)$ and $\varphi_{3}\left(e_{H_{k,i}^{2}}\right)$, the generalized M-estimation based criterion can be obtained by:

(34) $$\begin{array}{l} P_{k|k-1}^{-1}r_{x_{k|k-1}}+\sum_{i=1}^{n_{z}}R_{i}^{-1}\left[\left(\frac{\partial e_{b_{k,i}}}{\partial {\hat{x}}_{k|k}}\right)\varphi_{1}\left(w_{bi}e_{b_{k,i}}\right)+\right.\\ \left.\left(\frac{\partial e_{H_{k,i}^{1}}}{\partial {\hat{x}}_{k|k}}\right)\varphi_{2}\left(w_{bi}e_{H_{k,i}^{1}}\right)+\left(\frac{\partial e_{H_{k,i}^{2}}}{\partial {\hat{x}}_{k|k}}\right)\varphi_{3}\left(w_{bi}e_{H_{k,i}^{2}}\right)\right]=0 \end{array}$$

Crucially, under the TLS framework, the effects of outliers in the observation vector $b_{k}$ and the coefficient matrix $H_{k}$ are coupled. Since $e_{H_{k,i}^{1}}$, $e_{H_{k,i’}^{1}}$, and $e_{b_{k,i}}$ all originate from the bearing error, the same weights $w_{bi}$ ($i=1,2,\cdots ,n_{z}$) are assigned to each measurement channel. When outliers are present, these weights are reduced, thereby attenuating the influence of outliers. It is noted that the effects of bearing outliers are bounded, and hence $\varphi_{i}(\cdot )$ is also a bounded function.

Importantly, Equation (32) reduces to the LS-TLS criterion when $\rho \left(e_{b_{k,i}},e_{H_{k,i}}\right)=$$e_{b_{k}}^{T}{\tilde{P}}_{b_{k}}^{-1}e_{b_{k}}+e_{H_{k}}^{T}{\tilde{P}}_{H_{k}}^{-1}e_{H_{k}}$. Substituting this expression into Equation (32) and taking the derivative yields:

(35) $$\begin{array}{l} P_{k|k-1}^{-1}r_{x_{k|k-1}}+\sum_{i=1}^{n_{z}}R_{i}^{-1}\left[\left(\frac{\partial e_{b_{k,i}}}{\partial {\hat{x}}_{k|k}}\right){\tilde{P}}_{b_{k}}^{-1}e_{b_{k,i}}+\left(\frac{\partial e_{H_{k,i}^{1}}}{\partial {\hat{x}}_{k|k}}\right)\left(e_{H_{k,i}^{1}}\sin^{2}{\tilde{z}}_{k,i}+\right.\right.\\ \left.e_{H_{k,i}^{2}}\sin{\tilde{z}}_{k,i}\cos{\tilde{z}}_{k,i}\right)+\left.\left(\frac{\partial e_{H_{k,i}^{2}}}{\partial {\hat{x}}_{k|k}}\right)\left(e_{H_{k,i}^{2}}\cos^{2}{\tilde{z}}_{k,i}+e_{H_{k,i}^{1}}\sin{\tilde{z}}_{k,i}\cos{\tilde{z}}_{k,i}\right)\right]=0 \end{array}$$

By comparing Equations (33) and (35), it can be seen that:

$$\varphi_{1}\left(e_{b_{k,i}}\right)={\tilde{P}}_{b_{k}}^{-1}e_{b_{k,i}}$$

$$\varphi_{2}\left(e_{H_{k,i}^{1}}\right)=e_{H_{k,i}^{1}}\sin^{2}{\tilde{z}}_{k,i}+e_{H_{k,i}^{2}}\sin{\tilde{z}}_{k,i}\cos{\tilde{z}}_{k,i}$$

$$\varphi_{3}\left(e_{H_{k,i}^{2}}\right)=e_{H_{k,i}^{2}}\cos^{2}{\tilde{z}}_{k,i}+e_{H_{k,i}^{1}}\sin{\tilde{z}}_{k,i}\cos{\tilde{z}}_{k,i}$$

Accordingly, Equation (34) can be rewritten as:

(36) $$\begin{array}{l} P_{k|k-1}^{-1}r_{x_{k|k-1}}+\sum_{i=1}^{n_{z}}{\bar{w}}_{k,i}\left[\left(\frac{\partial e_{b_{k,i}}}{\partial {\hat{x}}_{k|k}}\right){\tilde{P}}_{b_{k}}^{-1}e_{b_{k,i}}+\left(\frac{\partial e_{H_{k,i}^{1}}}{\partial {\hat{x}}_{k|k}}\right)\left(e_{H_{k,i}^{1}}\sin^{2}{\tilde{z}}_{k,i}+\right.\right.\\ \left.e_{H_{k,i}^{2}}\sin{\tilde{z}}_{k,i}\cos{\tilde{z}}_{k,i}\right)+\left.\left(\frac{\partial e_{H_{k,i}^{2}}}{\partial {\hat{x}}_{k|k}}\right)\left(e_{H_{k,i}^{2}}\cos^{2}{\tilde{z}}_{k}+e_{H_{k,i}^{1}}\sin{\tilde{z}}_{k,i}\cos{\tilde{z}}_{k,i}\right)\right]=0 \end{array}$$

which is called the generalized M-estimation criterion, where ${\bar{w}}_{k,i}=R_{i}^{-1}w_{bi}$ can be interpreted as an equivalent weight replacing the prior weight $R_{i}^{-1}$.

Therefore, the only difference between the robust criterion (36) and the Gaussian criterion is the introduction of the weight matrix ${\bar{w}}_{k}=\mathrm{diag}\left({\bar{w}}_{k,i}\right)$. This implies that the robust solution can be obtained within the standard SCKF framework by replacing $R^{-1}$ with ${\bar{w}}_{k}$, i.e., without altering the overall filtering structure.

The partial derivatives $\partial e_{b_{k,i}}/\partial {\hat{x}}_{k|k}$, $\partial e_{H_{k,i}^{1}}/\partial {\hat{x}}_{k|k}$, and $\partial e_{H_{k,i}^{2}}/\partial {\hat{x}}_{k|k}$ appearing in Equations (33)–(36) are used solely in the theoretical analysis to derive the equivalent weight matrix ${\bar{w}}_{k}$. In the actual RSCKF algorithm, these Jacobian evaluations are never computed.

Specifically, in the SCKF framework, the innovation square-root factor ***S***_*zz*,*k*|*k*−1_ is computed by a QR-based factor update, as shown in Equation (15). In the proposed RSCKF, the square-root recursion is not changed. The only modification is that the original measurement weight is replaced by the equivalent weight ${\bar{w}}_{k}$, and the corresponding equivalent measurement-noise square-root factor ${\bar{S}}_{k}$ is used in the QR update. Thus, Equation (15) can be reformulated as:

(37) $${\bar{S}}_{zz,k|k-1}=\mathrm{qr}\left({\left[\zeta_{k|k-1},{\bar{S}}_{R}\right]}^{T}\right)$$

where ${\bar{S}}_{zz,k|k-1}$ is an equivalent square-root factor of equivalent innovation covariance, and ${\bar{w}}_{k}^{-1}={\bar{S}}_{R}{\bar{S}}_{R}^{T}$. Therefore, Equation (37) preserves the QR-based square-root structure of the standard SCKF. The cubature-point generation, the triangular system solution used in the Kalman gain computation, and the square-root covariance update remain unchanged. When $\bar{w}$_*k*,*i*_ = 0, the corresponding scalar measurement update is skipped rather than implemented by explicitly inverting a zero weight. Hence, the estimated state ${\hat{x}}_{k|k}$ and the corresponding $S_{k|k}$ can be obtained by:

(38) $${\hat{x}}_{k|k}={\hat{x}}_{k|k-1}+{\bar{K}}_{k}(z_{k}-{\hat{z}}_{k|k-1})$$

(39) $$S_{k|k}=\mathrm{qr}\left({\left[\chi_{k|k-1}-{\bar{K}}_{k}\zeta_{k|k-1},\;{\bar{K}}_{k}{\bar{S}}_{R}\right]}^{T}\right)$$

where the equivalent Kalman gain ${\bar{K}}_{k}$ is:

(40) $${\bar{K}}_{k}=\left(P_{xz,k|k-1}/{\left({\bar{S}}_{zz,k|k-1}\right)}^{T}\right)/{\bar{S}}_{zz,k|k-1}$$

This derivation demonstrates that combining M-estimation with the SCKF under the TLS criterion can be achieved by simply modifying the measurement noise covariance based on the weights ${\bar{w}}_{k}$. Compared with conventional robust algorithms, the RSCKF solution offers two key advantages: (i) M-estimation and the SCKF are integrated without linearization of the measurement equation, allowing both accuracy and robustness to be fully exploited. (ii) The solution framework of the RSCKF remains identical to that of the SCKF, thereby preserving the derivative-free nature and numerical stability of the original SCKF while providing robustness against non-Gaussian outliers modeled by Equation (20). The equivalent-weighting idea can be extended to other nonlinear filtering problems when a meaningful innovation-based reliability statistic and an equivalent covariance interpretation are available. However, the TLS-based motivation in this paper is specifically tied to the pseudo-linear errors-in-variables structure of the BOT measurement model.

The analysis above shows that robustness can be incorporated into the SCKF by replacing $R^{-1}$ with an equivalent weight matrix ${\bar{w}}_{k}$. The specific form of ${\bar{w}}_{k,i}$ depends on the choice of the score function ρ(⋅). Rather than selecting a particular ρ(⋅) and solving for the corresponding weight (which typically requires iterations), in the next section, we directly design a closed-form, non-iterative weight function based on a standardized innovation statistic, guided by the principle that the weight should reduce to the nominal value $R_{i}^{-1}$ for clean measurements, decrease for moderate outliers, and vanish for extreme ones.

## 4. Equivalent Weight Function Based on the Standard Euclidean Distance

According to the robust LS-TLS extremal function, handling outliers is equivalent to designing the weight function ${\bar{w}}_{k}$. A well-designed ${\bar{w}}_{k}$ can effectively reduce the weights assigned to outliers, thereby suppressing their influence. The design of ${\bar{w}}_{k}$ generally involves two components: an outlier judgment threshold and the corresponding weight assignment for the identified outliers.

Huber’s original weight function is given by [7]:

(41) $${\bar{w}}_{k,i}=\left\{\begin{array}{ll} R_{i}^{-1} & \left|{\tilde{r}}_{k,i}\right|\leq c\;\\ \mathrm{sgn}\left({\tilde{r}}_{k,i}\right)\frac{R_{i}^{-1}\cdot c}{{\tilde{r}}_{k,i}} & \;\left|{\tilde{r}}_{k,i}\right|>c \end{array}\right.$$

where $R_{i}^{-1}$ represents the prior weight, ${\tilde{r}}_{k,i}$ is the *i*-th standard residual, and c is the judgment threshold [35]. When an outlier occurs, Huber’s weight function reduces the weight according to the standard residual. However, several drawbacks can be identified in Equation (41). Firstly, the outlier judgment threshold c, which is based on the residual, is typically empirical due to the complicated sources of errors in ${\tilde{r}}_{k,i}$. Secondly, large outliers may still affect the stability of the filter because Huber’s weight function only decreases linearly. Thirdly, iterations are required to compute the equivalent weights, which may result in poor computational efficiency.

To address these problems, the standardized Euclidean distance between the actual measurement $z_{k,i}$ and the predicted measurement ${\hat{z}}_{k|k-1,i}$ is introduced to identify outliers. In the scalar bearing measurement setting considered in this paper, the innovation covariance reduces to a scalar, and the standardized innovation is straightforwardly defined as:

(42) $$\lambda_{k,i}=\left(z_{k,i}-{\hat{z}}_{k|k-1,i}\right)/S_{zz,k|k-1,i}$$

where $S_{zz,k|k-1,i}$ is the *i*-th diagonal component of $S_{zz,k|k-1}$.

The variable $\lambda_{k,i}$ approximately follows a standard Gaussian distribution when process noise and measurement noise are both Gaussian distributed with zero mean. Let $\gamma_{\alpha }$ denote the α- quantile, where α is the significance level. The probability $p\left(\left|\lambda_{k,i}\right|>\gamma_{\alpha }\right)$ is then given by:

(43) $$p\left(\left|\lambda_{k,i}\right|>\gamma_{\alpha }\right)=\alpha$$

If $\left|\lambda_{k,i}\right|>\gamma_{\alpha }$, the corresponding measurement can be regarded as being contaminated by an outlier. That is, $\lambda_{k,i}$ will generally deviate from the standard Gaussian distribution when an outlier is present. Therefore, the judgment threshold can be determined based on the significance level α. Since the test is two-sided, $\gamma_{\alpha }$ equals the upper α/2-quantile of the standard normal distribution. For example, $\alpha_{1}=5\%$ yields $\gamma_{\alpha_{1}}=z_{0.025}=1.96$. The two confidence levels have different practical roles. The first level, $\alpha_{1}$, determines when a measurement begins to be treated as suspicious and hence controls the onset of down-weighting. The second level, $\alpha_{2}$, corresponds to a much rarer tail event and determines when the measurement is completely rejected. Therefore, the thresholds should be understood as statistically interpretable operating points of the normalized-innovation test, rather than as universal constants that are optimal for every tracking scenario. Their numerical influence is examined in the sensitivity analysis in Section 5.4.

In essence, the standardized Euclidean distance quantifies the inconsistency between the actual and the predicted measurement at each time step. When the noise is Gaussian, $\lambda_{k,i}$ approximately follows a Gaussian distribution, and $\left|\lambda_{k,i}\right|$ is typically smaller than the judgment threshold. Conversely, when an outlier occurs, $\lambda_{k,i}$ becomes abnormally large, enabling outlier identification. Although outliers can also be detected using other metrics such as the Mahalanobis distance [36], the Mahalanobis distance provides only an overall measure of discrepancy between the predicted and actual measurement vectors at a given time step. Applying a uniform weight factor to all measurement channels could improperly degrade the efficiency of uncontaminated measurements.

To ensure the effectiveness of the robust method, when an outlier is down-weighted by ${\bar{w}}_{k}$, the new judgment variable $\left|{\bar{\lambda }}_{k,i}\right|$ should not exceed the threshold $\gamma_{\alpha_{1}}$, i.e.,:

(44) $$\left|{\bar{\lambda }}_{k,i}\right|=\left|z_{k,i}-{\hat{z}}_{k|k-1,i}\right|/{\bar{S}}_{zz,k|k-1,i}\leq \gamma_{\alpha_{1}}$$

For the standard CKF, the innovation covariance matrix $S_{zz,k|k-1}S_{zz,k|k-1}^{T}$ can be written as:

(45) $$S_{zz,k|k-1}S_{zz,k|k-1}^{T}=\frac{1}{2n_{x}}\sum_{j=1}^{2n_{x}}\left(Z_{j,k|k-1}-{\hat{z}}_{k|k-1}\right){\left(Z_{j,k|k-1}-{\hat{z}}_{k|k-1}\right)}^{T}+R$$

Replacing prior covariance R with ${\bar{w}}_{k}^{-1}$, the equivalent innovation covariance matrix of the RSCKF can be obtained as:

(46) $${\bar{S}}_{zz,k|k-1}{\bar{S}}_{zz,k|k-1}^{T}=\frac{1}{2n_{x}}\sum_{j=1}^{2n_{x}}\left(Z_{j,k|k-1}-{\hat{z}}_{k|k-1}\right){\left(Z_{j,k|k-1}-{\hat{z}}_{k|k-1}\right)}^{T}+{\bar{w}}_{k}^{-1}$$

Substituting (45) and (46) into (44) yields:

(47) $${\bar{w}}_{k,i}^{-1}\geq \frac{\lambda_{k,i}^{2}S_{zz,k|k-1,i}S_{zz,k|k-1,i}^{T}}{\gamma_{\alpha_{1}}^{2}}-S_{zz,k|k-1,i}S_{zz,k|k-1,i}^{T}+R_{i}$$

Equation (47) provides a lower bound on the equivalent inverse weight ${\bar{w}}_{k,i}^{-1}$. Choosing the equality corresponds to the minimum-intervention principle. The weight is reduced just enough to bring the re-standardized innovation $\left|{\bar{\lambda }}_{k,i}\right|$ to the threshold $\gamma_{\alpha_{1}}$, thereby minimizing unnecessary information loss from down-weighting. Any larger ${\bar{w}}_{k,i}^{-1}$ would further inflate the equivalent noise covariance beyond what is needed for outlier suppression.

When a large outlier appears, $z_{k}$ deviates significantly from the true measurement, making the filter prone to divergence. Hence, the measurement update is rejected when $\left|\lambda_{k,i}\right|>\gamma_{\alpha_{2}}$, where $\gamma_{\alpha_{2}}$ is the extreme $\alpha_{2}$-quantile. In this case, ${\hat{x}}_{k|k}$ and $S_{k|k}$ are directly set as:

(48) $${\hat{x}}_{k|k}={\hat{x}}_{k|k-1}$$

(49) $$S_{k|k}=S_{k|k-1}$$

Therefore, combining these conditions, the three-piecewise equivalent weight function ${\bar{w}}_{k,i}$ is expressed as:

(50) $${\bar{w}}_{k,i}=\left\{\begin{array}{l} R_{i}^{-1}\;\;\;\;\;\;\;\;\;\;\;\;\;\;\;\;\;\;\;\left|\lambda_{k,i}\right|\leq \gamma_{\alpha_{1}}\;\\ {\left(\lambda_{k,i}^{2}S_{zz,k|k-1,i}S_{zz,k|k-1,i}^{T}/\gamma_{\alpha_{1}}^{2}-S_{zz,k|k-1,i}S_{zz,k|k-1,i}^{T}+R_{i}\right)}^{-1}\;\gamma_{\alpha_{1}}<\left|\lambda_{k,i}\right|\leq \gamma_{\alpha_{2}}\\ 0\;\;\;\;\;\;\;\;\;\;\;\;\;\;\;\;\;\;\;\;\left|\;\lambda_{k,i}\right|>\gamma_{\alpha_{2}} \end{array}\right.$$

Compared with Equation (41), the outlier judgment threshold of the proposed ${\bar{w}}_{k,i}$ is naturally determined based on the confidence level of $\lambda_{k,i}$, thereby reducing reliance on scenario-dependent empirical threshold selection. Additionally, no iterations are required in Equation (50), and hence the computational efficiency is superior to that of Equation (41). Furthermore, rejecting large outliers reduces the computational cost and protects both the estimated state and the square-root factor of the estimated covariance from adverse effects.

When multiple bearings are available simultaneously ($n_{z}>1$), the equivalent weight function in Equation (50) can be applied via a sequential scalar update strategy, where each bearing component is processed individually. This avoids the numerical difficulty of a partially singular equivalent noise covariance matrix that would arise if some channels are rejected (${\bar{w}}_{k,i}=0$) while others are retained in a vector update. Alternatively, one may form a reduced measurement vector by excluding rejected channels before performing the vector update. These implementation strategies are left for future investigation.

The complete RSCKF procedure is summarized in Algorithm 1.

**Algorithm 1.** Robust square-root cubature Kalman filter procedure.**Input:** ${\hat{x}}_{0|0}$$,\;S_{0|0}$, Q$,\;R=\mathrm{diag}\left(R_{i}\right)$$,\;\alpha_{1}$$,\;\alpha_{2}$$,\;\mathrm{measurements}\;\{z_{k}$} **Output:** $\;\mathrm{Filtered}\;\mathrm{state}\;\mathrm{estimates}\;{\hat{x}}_{k|k}$$\;\mathrm{and}\;\mathrm{square}\text{-}\mathrm{root}\;\mathrm{covariance}\;\mathrm{factors}\;S_{k|k}$**for** k=1,2,⋯,n **do**  *Time Update:*
    $\mathrm{Generate}\;\mathrm{and}\;\mathrm{propagate}\;\mathrm{cubature}\;\mathrm{points},\;\mathrm{then}\;\mathrm{obtain}\;\mathrm{the}\;\mathrm{predicted}\;\mathrm{state}\;{\hat{x}}_{k|k-1}$$\;\mathrm{and}\;\mathrm{the}\;\mathrm{predicted}\;\mathrm{covariance}\;S_{k|k-1}$ according to Equations (5)–(9).  *Measurement Update:*
    Regenerate cubature points and propagate through $h\left(\cdot \right)$$,\;\mathrm{then}\;\mathrm{compute}\;{\hat{z}}_{k|k-1}$$,\;S_{zz,k|k-1}$$,\;\mathrm{and}\;P_{xz,k|k-1}$ according to Equations (10)–(16)  **for** 
$i=1,2,\cdots ,n_{z}$
**.**
    $\mathrm{Compute}\;\mathrm{standardized}\;\mathrm{innovation}\;\lambda_{k,i}$ according to Equation (42).   
$\mathrm{Compute}\;{\bar{w}}_{k,i}$
$\;\mathrm{according}\;\mathrm{to}\;\mathrm{Equation}\;(50)\;\mathrm{based}\;\mathrm{on}\;\gamma_{\alpha_{1}}$
$\;\mathrm{and}\;\gamma_{\alpha_{2}}$
  **if** ${\bar{w}}_{k,i}=0$, **then**    skip the *i*-th scalar measurement update.  **else**
   
$\mathrm{perform}\;\mathrm{the}\;\mathrm{scalar}\;\mathrm{SCKF}\;\mathrm{measurement}\;\mathrm{update}\;\mathrm{using}\;\mathrm{the}\;\mathrm{equivalent}\;\mathrm{covariance}\;\mathrm{induced}\;\mathrm{by}\;{\bar{w}}_{k,i}$

  **end if**

**end for**


## 5. Numerical Experiments

In this section, the performance of seven filtering algorithms is compared: the standard CKF, the SCKF, the novel robust UKF (NRUKF) [12], the maximum correntropy criterion SCKF (MCCSCKF) [19], the outlier-detecting CKF (ODCKF) [26], the robust Student’s *t* CKF (RSTCKF) [22], and the proposed RSCKF. All algorithms share the same system model and initialization to ensure a fair comparison.

To describe the accuracy of the filters, the root mean square errors in position and velocity (RMSE_pos_, RMSE_vel_) are defined as in Equations (51) and (52), and the mean squared error in position (MSE_pos_) for each Monte Carlo run is defined as in Equation (53). The number of Monte Carlo runs is L=200.

(51) $$RMSE_{\mathrm{pos},k}=\sqrt{\frac{1}{L}\sum_{j=1}^{L}{\left({\hat{x}}_{k|k,j}^{t}-x_{k,j}^{t}\right)}^{2}+{\left({\hat{y}}_{k|k,j}^{t}-y_{k,j}^{t}\right)}^{2}}$$

(52) $$RMSE_{vel,k}=\sqrt{\frac{1}{L}\sum_{j=1}^{L}{\left({\hat{\dot{x}}}_{k|k,j}^{t}-{\dot{x}}_{k,j}^{t}\right)}^{2}+{\left({\hat{\dot{y}}}_{k|k,j}^{t}-{\dot{y}}_{k,j}^{t}\right)}^{2}}$$

(53) $$MSE_{\mathrm{pos},j}=\frac{1}{n}\sum_{k=1}^{n}\left[{\left({\hat{x}}_{k|k,j}^{t}-x_{k,j}^{t}\right)}^{2}+{\left({\hat{y}}_{k|k,j}^{t}-y_{k,j}^{t}\right)}^{2}\right]$$

The BOT system model given in Equations (3) and (4) is adopted to verify the effectiveness of the proposed RSCKF. A coordinated turn (CT) model is employed for the target dynamics, and a single maneuvering observer provides bearings-only measurements [37]. The target state is $x={\left[x,y,\dot{x},\dot{y}\right]}^{T}$, representing position and velocity. The CT model with a constant turn rate ω=3°/min is used as the state transition function, with the sampling period T=1min and the total number of time steps n=40. The process noise intensity is $q=1\times 10^{-4}{\mathrm{km}}^{2}{/\min}^{3}$. A single observer is deployed at the initial position ${\left[0,0\right]}^{T}km$. To ensure observability, the observer performs a maneuver. During K=1–20, it moves with velocity ${\left[0.15,-0.25\right]}^{T}\mathrm{km}/\min$; During k=21–40, it switches to ${\left[0.30,-0.05\right]}^{T}\mathrm{km}/\min$. The bearing measurement is given by Equation (4), and the standard deviation of the measurement noise is $\sigma_{z}=0.01\;rad$.

The true initial state is $x_{0}={\left[50,50,-0.3,-0.1\right]}^{T}$. The initial state estimate is set with biases: $\Delta x={\left[2,2,0.02,0.02\right]}^{T}$, and the initial error covariance is $P_{0}=\mathrm{diag}(4,4,0.04,0.04)$. All filters share the same initialization. To model measurement outliers, the Gaussian contaminated distribution in Equation (20) is adopted. Three cases are considered to evaluate performance under different contamination levels. In Case 1, the measurement noise follows the standard Gaussian distribution. In Cases 2 and 3, approximately 15% of the measurements are contaminated by outliers whose standard deviations are $10\sigma_{z}$ and $30\sigma_{z}$, respectively. The algorithm parameters are shown in Table 1.

In the proposed RSCKF, the significance levels $\alpha_{1}=5\%$ and $\alpha_{2}=0.1\%$ correspond to the quantiles $\gamma_{\alpha_{1}}=1.96$ and $\gamma_{\alpha_{2}}=3.29$, respectively. These thresholds are derived from the standard Gaussian distribution based on Equation (43), thereby reducing reliance on scenario-dependent empirical tuning. The parameters for the comparison algorithms are selected according to their original references.

### 5.1. Performance Comparison Without Measurement Outliers

**Figure 1:**
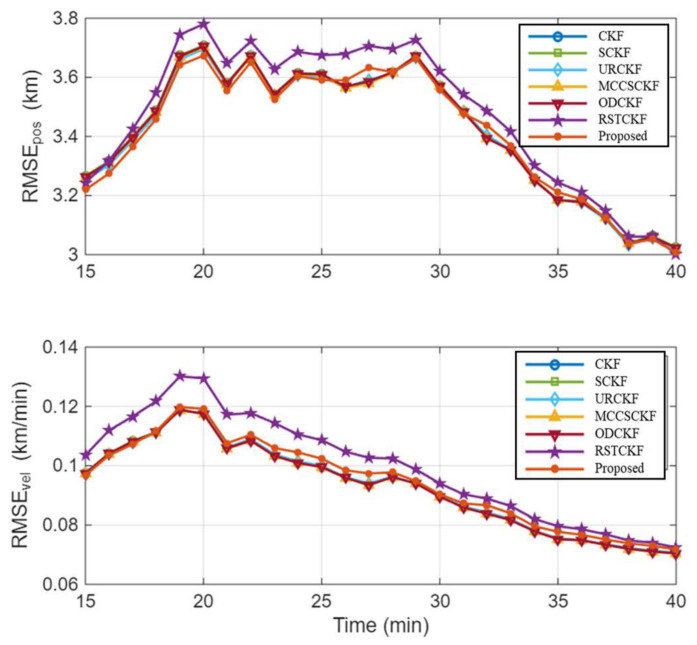
The RMSEs of algorithms when no outliers appear.

Without measurement outliers, the RMSE_pos_ and the RMSE_vel_ of the various filtering algorithms can be described in Figure 1, where “proposed” represents “the proposed RSCKF”.

As shown in Figure 1, all algorithms converge as the filter accumulates information from the maneuvering observer. Among the robust filters, the proposed RSCKF achieves MSE_pos_ of 10.42 km^2^, which is the lowest among all seven algorithms and marginally better than the CKF/SCKF. Remarkably, the RSCKF not only avoids performance loss compared to the baseline filters but also achieves slightly better accuracy, which can be attributed to the natural self-regularization effect of the three-piecewise weight function on occasional large normal-distribution tail events.

The NRUKF, MCCSCKF, and ODCKF perform very close to the baseline, indicating that these robust mechanisms introduce minimal perturbation under Gaussian noise. The RSTCKF exhibits a slightly higher MSE_pos_ of 10.70 km^2^ with noticeably larger variance, reflecting that its variational Bayesian iteration with Student’s *t* noise model may introduce additional estimation uncertainty even in the absence of outliers.

### 5.2. Performance Comparison with Moderate Outliers

**Figure 2:**
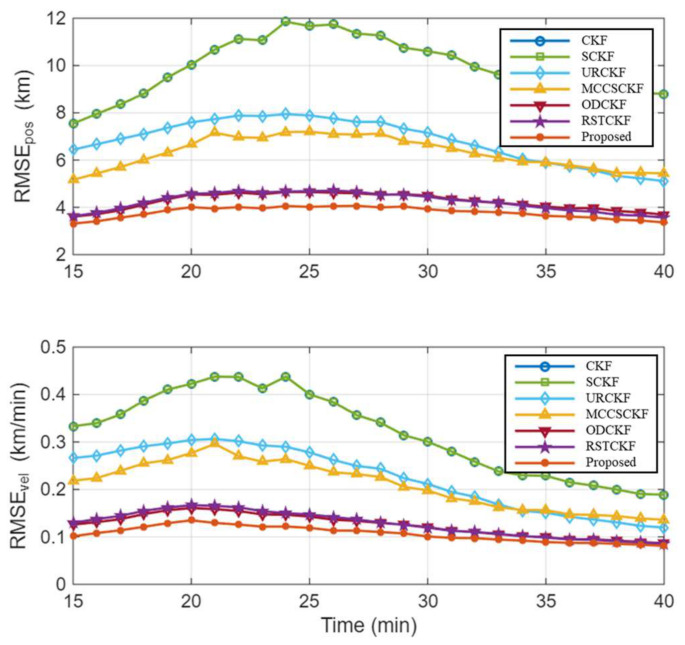
The RMSEs of algorithms with moderate outliers.

In Case 2, approximately 15% of the bearing measurements are drawn from N(0,100R), producing outliers with 10$\sigma_{z}$ standard deviation. The RMSE results are shown in Figure 2, and the MSE_pos_ values are summarized in Table 2.

Under moderate contamination, the non-robust CKF and SCKF degrade substantially, confirming their complete vulnerability to outliers. Among the robust algorithms, the proposed RSCKF achieves the best performance in both RMSE_pos_ and RMSE_vel_. Its MSE_pos_ represents only a 17% increase from the outlier-free case, demonstrating exceptional robustness. Two factors account for this superiority. First, the TLS-based extremal function enables the M-estimation to be introduced into the SCKF framework naturally without linearization or regression, fully preserving the derivative-free accuracy of the cubature rule. Second, the three-region equivalent weighting scheme detects outliers using a standardized innovation statistic with confidence-level thresholds. This enables principled down-weighting of moderate outliers and an outright rejection of extreme ones.

The ODCKF and RSTCKF achieve comparable performance through their Bernoulli mixture model and variational Bayesian approach, respectively, but remain inferior to the RSCKF because they do not exploit the TLS structure of the BOT measurement model. The MCC-SCKF shows limited effectiveness due to its inherent sensitivity to the kernel bandwidth. No single value is optimal across different contamination levels. In contrast, the thresholds in the RSCKF are statistically determined from the confidence level without scenario-dependent tuning. The NRUKF performs the worst among the robust filters, as its nonlinear regression procedure introduces additional linearization errors that the RSCKF inherently avoids by operating within the original SCKF solution framework.

### 5.3. Performance Comparison with Severe Outliers

**Figure 3:**
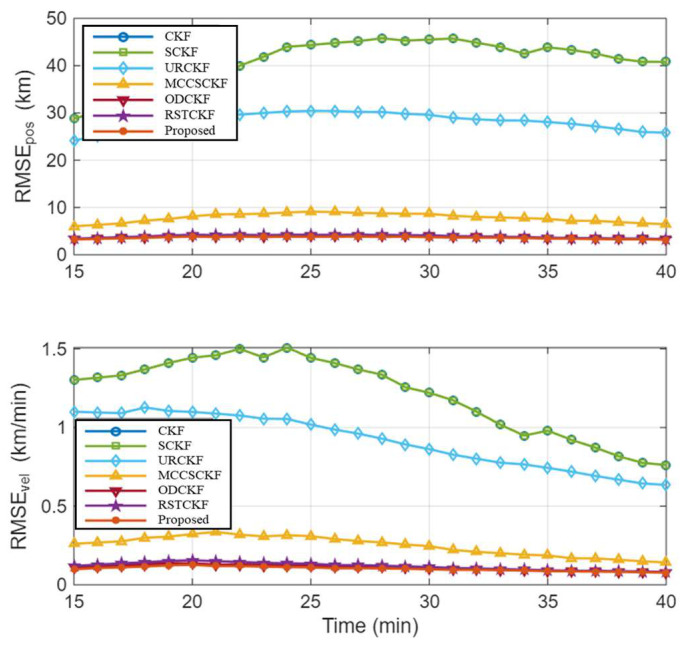
The RMSEs of algorithms with severe outliers.

In Case 3 (ε=0.15), outliers are drawn from $N\left(0,900R\right)$ with 30$\sigma_{z}$ standard deviation. The RMSE results are shown in Figure 3.

Under severe contamination, the proposed RSCKF demonstrates its clearest advantage. The CKF and SCKF completely fail, as they treat all measurements equally regardless of contamination. The NRUKF also degrades severely because the Huber weight decreases linearly but never reaches zero, so large outliers always exert residual influence on the state estimate.

In contrast, RSCKF maintains performance nearly identical to the outlier-free case. Since 30σ outliers almost always produce $\left|\lambda_{k,i}\right|\gg \gamma_{2}$, they are completely rejected via Equations (48) and (49), and the state and covariance are entirely shielded from contamination, as if the outlier measurement never occurred. The ODCKF and RSTCKF also maintain reasonable performance, where the former benefits from clearer outlier separation at higher κ, and the latter adaptively inflates the noise covariance through its heavy-tailed Student’s t model. However, neither achieves the RSCKF’s stability, because their soft-weighting mechanisms still allow partial outlier influence rather than eliminating it entirely. The MCC-SCKF improves considerably relative to Case 2, since the Gaussian kernel weight drops rapidly for large outliers, but the improvement is scenario-dependent and further confirms its sensitivity to the mismatch between kernel bandwidth and outlier magnitude.

### 5.4. Overall MSE and Computation Time Comparison

**Figure 4:**
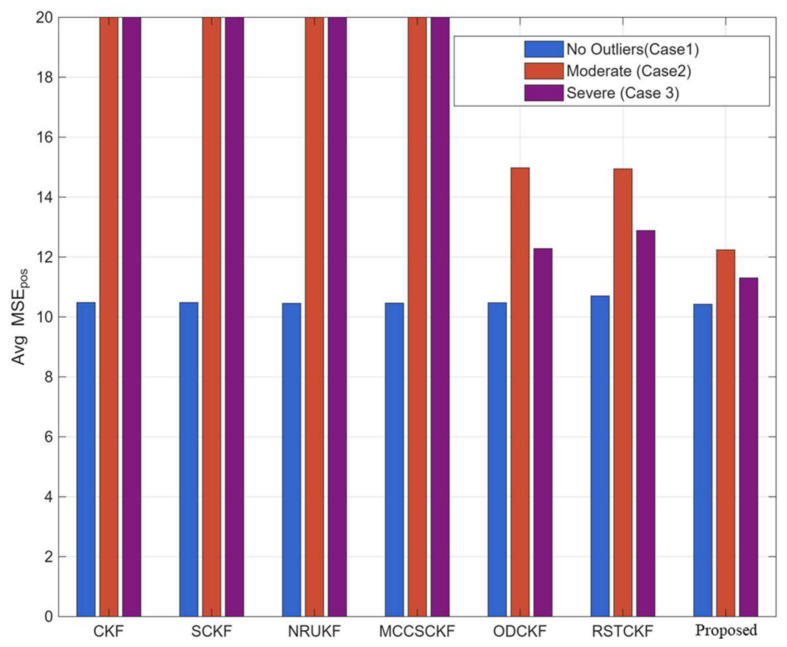
The average MSE_pos_ under different conditions.

The average MSE_pos_ and the corresponding variances across L=200 Monte Carlo runs are summarized in Table 2 and Figure 4.

Table 2 summarizes the average MSE_pos_ across the three contamination scenarios. The proposed RSCKF achieves the lowest MSE_pos_ in all cases and exhibits the smallest degradation from Case 1 to Case 3, with only an 8.4% increase. By comparison, the ODCKF and RSTCKF increase by 17% and 20%, respectively, and the SCKF increases by over two orders of magnitude. This result quantitatively confirms that the complete rejection mechanism of the RSCKF provides stronger protection than the soft-weighting strategies adopted by the other robust filters. Moreover, the RSCKF slightly outperforms the SCKF even in Case 1, which can be attributed to the mild regularization effect of the three-piecewise weight function on occasional large tail events of the nominal Gaussian noise.

This advantage can be interpreted from the pseudo-linear BOT model. After the bearing equation is transformed into the pseudo-linear form, a bearing error affects both the observation vector and the coefficient matrix, rather than appearing only as an additive residual. Therefore, a corrupted bearing may distort the update geometry as well as increase the innovation magnitude. The proposed TLS-motivated equivalent weighting is applied at the bearing-channel level, so the same weight attenuates the coupled error contribution associated with that bearing. The three-region rule further preserves nominal measurements, down-weights moderate outliers, and rejects extreme outliers before they enter the square-root update. In contrast, VB-based filters mainly rely on adaptive covariance inflation, while MCC-based filters mainly perform residual-level kernel reweighting. These mechanisms are effective general robustification tools, but they do not explicitly use the coupled errors-in-variables structure of the pseudo-linear BOT model. This explains why the proposed RSCKF achieves lower MSE and better ANEES consistency in Cases 2 and 3. A consistent filter should yield NEES values close to $n_{x}=4$. For L=200 Monte Carlo runs, the customary pointwise 95% Monte Carlo consistency band for NEES is [3.618, 4.401]. Table 3 reports the ANEES subsequently averaged over the steady-state interval $k\in \left[15,40\right]$ for compact comparison.

Under Case 1, all filters are consistent (ANEES ≈ 4). Under Case 2, the CKF/SCKF exhibits ANEES = 36.61, indicating severe covariance underestimation. Notably, the MCCSCKF, despite achieving reasonable MSE_pos_, produces ANEES = 14.43, revealing that its covariance estimate significantly underrepresents the true estimation uncertainty. Under Case 3, the proposed RSCKF achieves ANEES = 4.37, the only value remaining within this customary Monte Carlo consistency band among all seven filters, confirming that the RSCKF maintains both low estimation error and statistically consistent covariance estimates.

To examine whether the confidence-level thresholds require scenario-dependent manual tuning, the sensitivity of the RSCKF to $\alpha_{1}$ and $\alpha_{2}$ is examined by sweeping α_1_ ∈ {1%,2%,5%,10%} and α_2_ ∈ {0.01%,0.05%,0.1%,0.5%}. The performance is almost insensitive to α_2_, with less than 1% position-MSE variation across its entire range for any fixed α_1_. Under contaminated conditions, α_1_ has a moderate effect because it determines the onset of down-weighting; the MSE varies by approximately 18% in Case 2 and 11% in Case 3 as α_1_ ranges from 1% to 10%. Under Case 1, the entire parameter grid produces position-MSE values between 10.40 and 10.52, corresponding to a total variation of only 1.1%, which confirms negligible degradation under nominal Gaussian measurements. The recommended values α_1_ = 5% and α_2_ = 0.1% achieve the best nominal performance while remaining within 5% of the most aggressive setting under contamination. These results indicate that the recommended thresholds are not universal constants, but they provide statistically interpretable and practically stable operating points for the BOT scenarios considered in this study.

The relative computation times of the algorithms, normalized to the CKF, are presented in Table 4. Two scenarios are tested: normal measurements (Case 1) and a mixed scenario with 15% outliers at 30$\sigma_{z}$ intensity (Case 3).

As shown in Table 4, under nominal conditions (Case 1), the SCKF and RSCKF are only marginally slower than the CKF, whereas the iterative algorithms require substantially more time. The computational complexity of the standard SCKF is $O(n^{3})$ due to the QR decomposition and matrix operations. The proposed RSCKF introduces an additional step for calculating the weight matrix $w_{k}$, which involves scalar operations with complexity O(n). Therefore, the overall complexity of RSCKF remains $O(n^{3})$, preserving the efficiency of the standard SCKF. In contrast, iterative robust filters (e.g., NRUKF, RSTCKF) require multiple iterations per time step, leading to a complexity of $L\times O(n^{3})$, where L is the number of iterations. Under severe contamination (Case 3), the NRUKF and RSTCKF remain costly because their iterative procedures execute regardless of outlier magnitude, while the RSCKF drops to 1.05, approaching the CKF cost. This reduction is a direct consequence of the rejection mechanism in Equations (48) and (49), which bypasses the entire Kalman update for measurements, with $\left|\lambda_{k,i}\right|\gg \gamma_{2}$, saving the QR decomposition and gain computation at those time steps. The non-iterative nature of the RSCKF stems from the closed-form equivalent weight in Equation (47), which depends only on $\lambda_{k,i}$ available from the prediction step, ensuring a deterministic and bounded computation time per step. This deterministic, non-iterative computation is favorable for real-time bearings-only tracking applications with strict computational constraints, since the additional robustification only involves scalar innovation tests and closed-form weight evaluations. The main robustness–efficiency trade-off is controlled by the two confidence-level thresholds: more aggressive thresholds can reject outliers more effectively but may discard informative measurements, whereas more conservative thresholds preserve nominal efficiency but may leave residual outlier influence. Since the proposed weighting rule is closed-form, this trade-off does not introduce data-dependent inner iterations or a substantial increase in computational complexity.

## 6. Conclusions

This paper presented a non-iterative robust square-root cubature Kalman filter (RSCKF) for bearings-only tracking with measurement outliers. By exploiting the pseudo-linear EIV structure of the bearing model, M-estimation is integrated into the SCKF through a TLS-consistent equivalent weight matrix, preserving the derivative-free computation and square-root numerical stability without inner iterations. A three-piecewise weight function with statistically determined thresholds enables principled down-weighting of moderate outliers and complete rejection of extreme ones. The key feature of the proposed method is that this robustness is introduced without changing the QR-based square-root recursion of the SCKF. Monte Carlo simulations show that, under severe 15% contamination with $30\sigma_{z}$ bearing outliers, the proposed RSCKF increases its position MSE by only 8.4% relative to the nominal case, whereas the standard SCKF deteriorates by more than two orders of magnitude. The RSCKF also achieves the lowest position MSE among the seven compared filters and is the only method whose time-averaged ANEES remains within the customary Monte Carlo consistency band. Its normalized computation time remains close to the CKF baseline, indicating that the improved robustness is obtained with only a small computational overhead.

These results suggest that the proposed RSCKF is suitable for real-time passive tracking applications in which intermittent angular outliers may occur, such as passive sonar tracking, UAV bearings-only navigation, and related sensor-fusion systems. From a broader estimation perspective, the method can also be viewed as a covariance-aware nonlinear observer with explicit innovation-based outlier rejection. Compared with deterministic observers, such as classical state observers or ESOs in ADRC, it additionally provides covariance propagation and innovation-based measurement gating, although it requires a stochastic model and noise covariance information. Future work will focus on validation with real bearings-only datasets, such as passive sonar and UAV bearings-only navigation data, adaptive online selection of the confidence thresholds, and extension to asynchronous multi-sensor bearings-only tracking.

## Figures and Tables

**Table 1:** Algorithm parameters.

Algorithm	Key Parameters
CKF	Standard cubature Kalman filter
SCKF	Square-root version of CKF
NRUKF	Huber threshold c = 1.96, κ = 0
MCCSCKF	Kernel bandwidth $\sigma_{k}$ = 10$\sigma_{z},$ max iter = 5
ODCKF	$a_{0}$ = 0.1, $b_{0}$ = 0.9, ρ = 100, max iter = 3
RSTCKF	$v_{p}$ = 30, $v_{m}$ = 4, max iter = 5
Proposed RSCKF	$\alpha_{1}$ = 5% ($\gamma_{\alpha_{1}}$ = 1.96), $\alpha_{2}$ = 0.1% ($\gamma_{\alpha_{2}}$ = 3.29)

**Table 2:** Average MSE_pos_ comparison.

Algorithm	MSE_pos_ (Case 1)	MSE_pos_ (Case 2)	MSE_pos_ (Case 3)
CKF	10.48	73.50	1195.10
SCKF	10.48	73.50	1195.10
NRUKF	10.45	38.77	611.91
MCCSCKF	10.46	31.38	47.55
ODCKF	10.47	14.98	12.28
RSTCKF	10.70	14.94	12.88
Proposed RSCKF	10.42	12.24	11.30

**Table 3:** Time-averaged ANEES over $k\in \left[15,40\right]$.

Filter	CKF	SCKF	NRUKF	MCCSCKF	ODCKF	RSTCKF	RSCKF
Case 1	4.20	4.20	4.20	4.19	4.20	4.08	4.23
Case 2	36.61	36.61	12.22	14.43	5.59	4.74	4.69
Case 3	840.01	840.01	150.13	14.56	4.72	4.44	4.37

**Table 4:** Comparison of relative computation times.

Scenario	CKF	SCKF	NRUKF	MCCSCKF	ODCKF	RSTCKF	RSCKF
Case 1	1.00	1.02	1.51	1.14	1.27	1.38	1.09
Case 3	1.00	1.07	1.54	1.11	1.38	1.44	1.05

## Data Availability

Dataset available on request from the authors.
